# Teachers’ perspectives on effective English language teaching practices at the elementary level: A phenomenological study

**DOI:** 10.1016/j.heliyon.2024.e29175

**Published:** 2024-04-07

**Authors:** Muhammad Imran, Norah Almusharraf, Mohamed Sayed Abdellatif, Abdul Ghaffar

**Affiliations:** a Education Research Lab, Prince Sultan University, Saudi Arabia; b Department of English, The University of Sahiwal, Pakistan; c Department of Linguistics and Translation, College of Humanities and Sciences, Prince Sultan University, Saudi Arabia; d Department of Educational Sciences, College of Education in Al-Kharj, Prince Sattam Bin Abdulaziz University, Saudi Arabia; e Department of Educational Psychology, College of Education in Assiut, Al-Azhar University, Egypt; f Department of English, University of Education, Multan Campus, Pakistan

**Keywords:** Teachers' professional development, Instructional practices, English language teaching, Challenges in language education

## Abstract

This study examined instructional practices and challenges English language teachers face in elementary schools. This study used a phenomenological approach and a mixed-method design. The data were collected through four tools: questionnaires, case studies, interviews, and observations in eight elementary schools in which eight educators and two hundred students participated from schools of three districts in central Punjab, Pakistan. This study aimed to explore the perspectives of teachers and students regarding the current pedagogical and instructional practices employed in English language classes. This study identified issues related to the lack of professional training and qualifications, overcrowded classrooms, cultural and social barriers, limited availability of the latest resources and technology, and a lack of parental cooperation. The findings suggested revisiting teachers’ professional development programs, focusing on innovative teaching methods, incorporating technology into language teaching classes and classroom materials development, and adaptation preparation. It further suggested that teachers with low levels of professional qualifications and training should consider focusing on specific approaches to meet the challenges they face in language classes instead of general teaching approaches.

## 1 Introduction

Changes in contemporary instructional practices, pedagogies, and teaching methods such as technology-based, inquiry-based, and problem-centered instructional approaches, and students’ centered encourage language teachers to revisit their traditional approaches to meet the challenges of the growing demand for English as a lingua franca [1,2]. Since the last decade, the Pakistani government has tried to implement significant changes in the educational system in response to global challenges by investing in quality education and resources to promote state-of-the-art English Language Teaching (ELT) at the grassroots level [3,4]. After the 18th amendment to the constitution of Pakistan in 2010, the Department of Education was devolved to the provincial level, and every province is responsible for its improvement and policy-making [5,6]. Similarly, in Punjab, the government emphasized enhancing the infrastructure of educational and vocational institutions along with a specific focus on English language learning and teaching due to the growing impact and demand in the local market and internationally [7].

The emphasis on English language teaching in Pakistan is due to the emergence of the English language as a *lingua franca* in official works, court language, businesses, technology, and competitive examinations for national and international jobs and higher education within and outside the country [8]. This is why English language teaching (ELT) receives significant focus in the school education department, as since the year 2010, in most public schools, English has been the medium of instruction from primary school to university education [1,9]. Similarly, other studies [8,10,11] discussed how the language of instruction significantly impacts learners' success in other subject areas taught in non-native languages, such as Urdu or Arabic-speaking children getting their primary education in English or French. However, in Pakistan, English language teaching needs more attention in public elementary schools in rural areas [11] because the provision of quality English language learning and teaching practices has been limited to elite public and private institutions, where access is limited to a small number of privileged families’ children [12]. As indicated by Refs. [11,13], the population in rural areas, particularly in the central-southern districts of Punjab, Pakistan, has no other choice except to attend low-quality public and private schools where most of the staff is not qualified, trained, and equipped with the latest ELT methodologies and technologies. Furthermore, this low-quality ELT situation persists throughout rural and semi-urban areas in the country [11,12].

The present study attempts to provide evidence from classroom practices to discuss current instructional practices used in English language teaching at the elementary level and the challenges to implementing them in elementary schools. In addition, this study highlights the gap in addressing the challenges, obstacles, and problems English teachers face during classroom practices of English teaching. Notably, the lack of technology integration and teachers' professional training is mainly focused on in this current study as most elementary school learners lack basic language learning training and struggle to cope with advanced studies in higher education and workplaces [11]. Hence, a solid foundation in primary language skills must be established for comprehensive language development among learners. Teachers' attitudes, teaching methods, approaches, and challenges play significant roles in purposefully disseminating English language teaching [9,12,13]. Against this backdrop, a critical question arises regarding the success and effectiveness of learning and teaching practices in public elementary schools, where teachers and students encounter numerous challenges, such as a lack of resources, technology integration, trained staff, and a friendly classroom environment. The findings will provide evidence from the ELT teachers’ classroom lessons and highlight to what extent ELT learning and teaching practices in elementary schools help address the issues related to substantive English language learning.

## 2 Literature review

Learning English is vital for developing countries like Pakistan to keep up with the growing challenges of the world. In Pakistan, English is taught as a compulsory subject from primary to graduation [11]. Moreover, English is the medium of instruction and teaching practices in both public and private schools [14]. Therefore, English language teaching (ELT) at the elementary level is crucial in setting the foundation for students' language development. This literature review aims to examine existing research on instructional practices and challenges in ELT at the elementary level. The aim is to gain insights into the phenomenological aspects of teaching English to young learners and identifies educators’ challenges in this context.

### 2.1 Importance of English language teaching in Pakistan

In the Pakistani context, English language competency is compulsory, as without it, it becomes challenging to excel in any sector, including the international market [9,15,16]. Therefore, numerous scholars have consistently emphasized instructional practices and challenges related to English language teaching to bring attention to the importance of English teaching in Pakistan among higher authorities and policymakers. For example [17], illustrates that English teaching in Pakistan faces many challenges and difficulties in meeting its standards, including the lack of capacity-building courses and programs for language teachers, inadequate infrastructure, overcrowded classrooms, reliance on a traditional examination system, teachers’ heavy workload, and insufficient professional qualifications.

### 2.2 Pedagogical approaches and strategies

Several studies, including [14,17,18], have examined the role of teachers in implementing language teaching policies, specifically English, in rural public schools in Pakistan. They argue that teaching English through the English medium policy yielded less favorable results because students did not demonstrate satisfactory performance in language learning classes. In addition [11,14], have highlighted the shortage of trained teachers, lack of structured pedagogical methods and techniques, lack of infrastructure, and socio-cultural dynamics in rural public schools in Pakistan.

### 2.3 Challenges in ELT at the school level

Furthermore [19,20], have examined the trends, issues, and challenges associated with English language teaching policies and practices in Pakistan. According to these studies, English language teaching and education in Pakistan face significant difficulties due to frequent policy shifts introduced by different governments. The primary challenge lies in determining the medium of instruction for both teachers and learners. However, several other issues have been highlighted, such as the shortage of trained and proficient English language teachers, inadequate provision of infrastructure and facilities, and the lack of adequate policy implementation in public schools in Pakistan.

Similarly [11,18], have brought attention to the issues and challenges Pakistani English language teachers face in integrating information and communication technologies (ICTs) into elementary school classrooms. These studies further discussed the impact of ICT availability and awareness on learners' and teachers' creativity and critical thinking abilities. Furthermore, they highlight the lack of professional training and qualifications among teachers in Pakistan, particularly in rural areas, which are necessary for effective English language instruction. In addition [10], asserted that students' performance is directly related to the influence of teachers' classroom performance on students' academic performance, as teachers' characteristics are reflected in students' personalities. Other studies [[21], [22], [23], [24], [25], [26], [27]] extensively examined various aspects related to English teaching in developing countries, specifically Pakistan. These studies explore instructional practices, attitudes, policies, infrastructure, and teacher's professional development, targeting multiple locations within Pakistan.

### 2.4 Teachers’ instructional practices and competence

Classroom instruction and educators' competence are the critical components of successful language teaching [28], particularly at the elementary level, where learners heavily rely on educators' experience and knowledge [10]. Furthermore [[29], [30], [31]], assert that teachers' practices and cognition are crucial in teaching English. They emphasize the importance of improving teachers' capacity through professional training and familiarizing them with technologies used worldwide for language teaching to enhance performance in English teaching. Similarly [32]) conducted a systematic review of ELT teachers’ engagement in the classroom and competence from 2010 to 2020. The review emphasized that ELT teachers require more training and professional development to deliver quality ELT teaching through modern technologies and interactive methodologies. However [33], highlights the significance of multimodal teaching practices in an ELT classroom, where teachers should utilize various designs and models to facilitate the language learning process.

## 3 Methodology

A mixed-method approach with a phenomenological orientation is employed to achieve the aims of this study. This phenomenological inquiry targets the individual's understanding of personal experiences of a specific situation, such as an ELT teacher's performance in the classroom. It examines the interpretation and application of their understanding, involvement, and description [34]. Initially, this study is limited to eight public schools from the three districts of central Punjab, Pakistan. According to Ref. [35], a mixed-method research approach is a comprehensive approach that incorporates both theoretical and empirical findings.

This study involved teachers and learners as volunteer participants for up to six weeks on different occasions for data collection through observations, questionnaires, and semi-structured interviews. English teachers from the selected public schools and their students were involved in this study to obtain comprehensive and in-depth results. The questionnaires were designed to be simple and understandable for the elementary students and the staff, ensuring they could fully comprehend the situation and respond efficiently. Occasionally, verbal Urdu translation was provided to aid students’ understanding of the English questionnaires. The interviews with the teachers typically lasted for 10–15 min and focused on their instructional practices, teaching challenges, and proposed solutions. The questionnaire and other data collection tools, such as interviews and observations, were utilized to gather data from eight selected public elementary schools. The data revealed the primary challenges and issues in instructional practices and overall English teaching in Punjab, Pakistan, as reported by the two hundred learners and eight teachers who participated in this study.

### 3.1 Data sampling

The researchers deliberately selected eight teachers and two hundred students from three districts of central Punjab, Pakistan. These schools were chosen randomly based on their location and provision of English language teaching classes from grades 1 to 8. The researcher requested the participation of one English teacher and twenty-five learners from each school as volunteers to investigate the instructional practices and learning issues. During a period of ten weeks, twenty-four classroom presentations and demonstrations were observed, with each English teacher conducting three. Additionally, interviews and interactive sessions were conducted, ranging from dialogue to semi-structured interviews with English language teachers. Furthermore, two hundred students, aged between 10 and 14 years, from these eight elementary schools were involved in this study through questionnaires and semi-structured interviews.

### 3.2 Observations

The traditional and convenient observation method was utilized as a part of the ethnography methodology to identify challenges and issues, as it is widely used in social sciences research. A random observation of twenty-four English lessons was conducted to investigate the challenges in instructional practices and teaching English as a foreign language. In the non-participant observation approach, the researchers observed and recorded practices without actively participating or providing any instruction or suggestion during the natural flow of the lessons. Each observation class lasted between thirty to 45 min. Field notes were taken according to the predetermined plan to collect, record, and compile data during the study. While this method did not directly answer the researchers’ questions, it was valuable for gathering information on challenges and issues in English language teaching and integrating instructional practices [36,37]. argue that “perceptions allow the researchers to assemble information on the scheduled and prescribed setting, the physical, i.e., human setting, and the interactional setting and the program setting” (p. 10). The researchers collected data by observing the physical settings of the participants and watching the responses of the individuals involved in the study. Table 1 below details English teachers, their lessons, and techniques noticed during classroom lesson observation.

**Table 1:** Lesson information during classroom observations.

Respondents	Subject	Lessons specification	Institution location	A.V. Aids
Respondent 1	ELT	Listening & Reading	Urban	Whiteboard, pointer, textbook, computer
Respondent 2	ELT	Grammar and vocabulary	Rural	Blackboard, chalk, and textbook
Respondent 3	ELT	Tenses	Urban	Whiteboard, pointer, textbook, computer
Respondent 4	ELT	Writing	Rural	Whiteboard, pointer, textbook,
Respondent 5	ELT	Speaking	Urban	Whiteboard, pointer, textbook, computer games, data projector
Respondent 6	ELT	Grammar	Rural	Whiteboard, pointer, textbook, computer
Respondent 7	ELT	Reading and Speaking	Urban	Whiteboard, pointer, textbook, computer
Respondent 8	ELT	Vocabulary and reading	Rural	Whiteboard, pointer, textbook,

### 3.3 Interviews

Eight teachers and forty randomly selected students were interviewed. Each interview lasted for 10–15 min. The interviews were conducted in a comfortable and cooperative manner; interview questions were in English. However, questions were also translated into Urdu for better understanding for some participants [38]. suggested that the researchers use a convenient interview format that allows respondents to remain flexible and willing to provide suggestions, describe challenges, and share experiences without obstacles. In these interviews, diverse questions, including open-ended and some close-ended questions, were used. The purpose of conducting face-to-face interviews with the respondents was to ensure the clarity of responses, which were expected and sought to be obtained from the participants, as the results and findings of the study are based on these outcomes. The interviewed teachers possessed various qualifications, age groups, and professional training. Table 2 shows the details of the English teachers interviewed for this study.

**Table 2:** Details of the teachers interviewed for this study.

Teacher name	Location: urban/rural	Age	Education	Professional qualifications
Respondent 1	Urban	34–38	MA in English	TEFL Diploma
Respondent 2	Rural	25–30	BS Honors	N/A
Respondent 3	Urban	40–45	MA Education, B. Ed	TEFL Diploma
Respondent 4	Rural	23–26	BS Honors	N/A
Respondent 5	Urban	45–50	B.A, B. Ed	TEFL Diploma
Respondent 6	Rural	43–47	BA, M. Ed	N/A
Respondent 7	Urban	30–35	M. A English	N/A
Respondent 8	Rural	31–36	MA Education, B. Ed	N/A

### 3.4 Data collection procedure

A consistent methodology was employed across all eight schools for data collection. Initially, the heads of the selected institutes were approached, either based on recommendations from local contacts or due to their demonstrated willingness during random visits before the study. Subsequently, all participants, including the heads of schools, English teachers, and students, were interviewed to obtain primary information about their qualifications, experiences, class/students, subject-related facilities, lesson planning, and any other relevant information for the observation. The researchers provided the participants with pertinent information about the study, including the purpose, classroom observation, and teachers' and learners’ involvement. Depending on the availability of English language teachers, at least one teacher and their three lessons were observed in each school, with detailed notes taken using a prepared performa. After the lesson observations, questionnaires and additional interviews were conducted with the teachers and learners. All the gathered information was then transferred into formal notes. To analyze the data, the researchers first filtered the results from the school observations, interviews, and questionnaires to identify the local challenges. Three thematic analysis techniques, contextual, narrative, and comparative, were applied to examine the different issues and challenges English teachers face at various locations. Subsequently, the researchers made their final recommendations regarding the matters in instructional practices and the challenges the selected English language teachers faced.

**Fig. 1:**
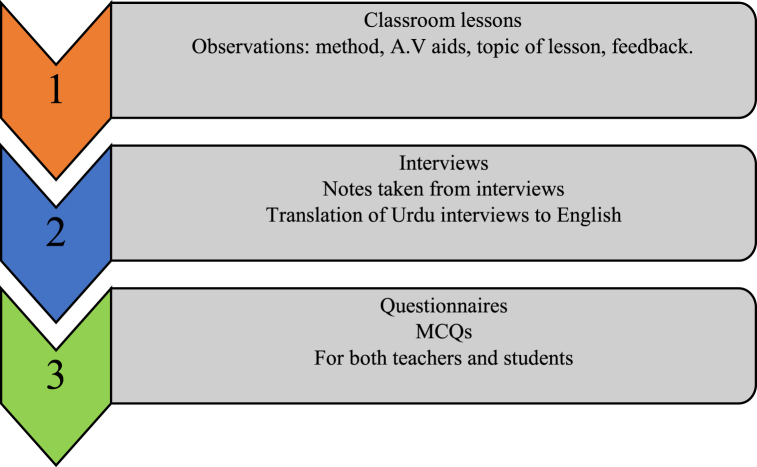
Flow of the data collection process.

The following Fig. 1 shows the flow of data collected during this study.

## 4 Findings and discussion

This part of the study presents the findings of the current study, conducted to discuss instructional practices and address the challenges English teachers face at elementary schools. The data collected highlight the phenomenological understanding of the importance of effective instructional practices that ELT teachers expressed in their interviews. In the following section of this study, the data collected via questionnaires, observation, and interviews have been discussed separately so that the readers can comprehensively understand the selected regions' ELT teachers' instructional practices, classroom challenges, and learners’ feedback.

### 4.1 Observation

Observation was employed as the primary method for data collection. Three classes were randomly selected from a pool of eight teachers for the observation process, and as a result, a total of twenty-four classes of 45 min were observed. Reactions were collected during the observation process and divided into sub-categories. Table 3 provides details of the observation process and its division into sub-categories as mentioned in the following.

**Table 3:** Observation process divided into sub-categories.

Tool	Reaction Topics	Thematic Analysis Technique
Observation	Instructors' preparation of lessons and their coordination with ELT	Contextual analysis, Narrative analysis
Observation	ELT abilities of educators	Comparative analysis
Observation	Encounters in ELT combination in the teaching of writing and reading	Narrative analysis, Comparative analysis
Observation	How the latest technology tools can be utilized to help ELT instructors	Pattern recognition, Narrative analysis
Observation	The distinction between utilizing the latest technology tools and instructing without utilizing them	Narrative analysis, Comparative analysis
Observation	To collect the relevant data about the issues and challenges the ELT teachers have faced during their classes	Pattern recognition, Narrative analysis, Contextual analysis
Observation	To document information regarding the issues and challenges of instructional practices.	Contextual analysis, Comparative analysis

The researchers acted as passive observers during the data collection process, ensuring no interruptions or interference with the lessons. They did not provide advice or instructions to the teachers or students. During the observation process, the researchers found many differences between trained and untrained teachers’ English language teaching methods, style, classroom management, and use of AV aids. However, the challenges in the classroom, instructional practices, and the facilities provided to the teachers and students for language learning have been examined thoroughly. Table 4 below provides an overview of the data collected during observation.

**Table 4:** Observation overview for data analysis themes.

	Item	Description of the items
1	Classroom management	Some issues about classroom management ability have been recorded between urban and rural ELT teachers. Lack of proficiency in subject matter also has been observed as a big challenge for ELT teachers.
2	Teaching Methods/Approaches	Usually, traditional teaching methods in urban and rural schools have been observed without innovation or creativity. However, few innovative and latest ELT teaching methods have been recorded in urban schools.
3	Availability of ELT Facilities	A considerable variation in the provision of facilities in ELT classrooms has been observed: urban schools have computer labs, while rural schools do not have such facilities.
4	Activities and Interactions in ELT Teaching	The biggest challenge for rural and urban ELT teachers is the language barrier while conducting any activity for English language teaching in classes. The students at the elementary level could not fully understand English instructions, so they used the local language most of the time to perform activities.
5	Other observed significant challenges faced by ELT teachers	There is a lack of professional training, an inability to use AV aids during teaching properly, and a lack of innovative activities and curriculum resources for ELT.

### 4.2 Professional development of ELT teachers

The primary objective of this inquiry was to investigate whether elementary school instructors received training in the integration of the latest technology and AV aids in their educational modules. In rural elementary schools, the respondents indicated that teachers received only partial training instead or had to prepare themselves for it. Conversely, among the respondents from urban schools, 40 % reported receiving training in the use of the latest technology and AV aids. However, 60 % of the instructors in densely populated urban institutions confirmed receiving comprehensive training in utilizing audiovisual aids effectively in their classrooms.

### 4.3 Innovations and technology-based activities in ELT classrooms

Newly inducted educators and teachers have received basic information and necessary training on utilizing the latest technology and AV aids in classrooms. However, most teachers and students acknowledged that they had never prepared for the specific techniques required to adapt to English learning in the classroom. However, in some schools, the teachers and students admitted that, to some extent, they are familiar with the latest techniques of the latest technology and AV aids.

**Fig. 2:**
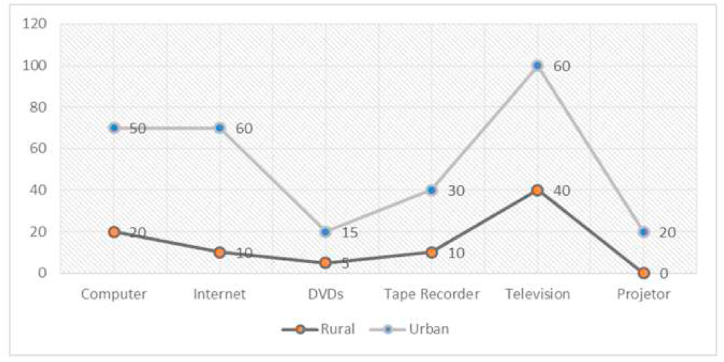
Skills chart for both rural and urban region institutions concerning the latest technology and AV aid utilization.

During the monitoring process, the researcher observed demonstrations and actual teaching lessons. None of the English educators integrated information and communication technology (ICT) into their teaching implementation. However, during discussions, they alluded to their ability and willingness to incorporate ICT, indicating they possessed the necessary skills, tools, and guidance. The following Fig. 2 shows how many facilities of the latest technology and AV aids are available for the teachers and how much they know about them.

### 4.4 Interviews with ELT teachers

Data collection through interviews with the teachers involved using pre-defined questions that were determined during the initial data collection phase. Furthermore, these interviews provided an opportunity for ELT teachers to share any additional challenges they have encountered in their teaching routines. The qualitative analysis was performed on the data collected from these teacher interviews. This qualitative analysis program facilitated finding the significant themes related to collected data, which were subsequently compiled thematically in the results. Table 5 provides details of each analysis theme and its description in the following.

**Table 5:** ELT teachers’ interviews analysis themes.

Sr.#	Analysis Themes	Description
1	ELT teaching challenges	Insufficient professional career-based training;
		Lack of proper infrastructure for quality ELT teaching;
		Overcrowded classrooms and overloaded teaching schedules.
2	Qualities of ELT teacher	Patience, ability to teach, subject knowledge;
		Regularity and punctuality;
		Eager to update themself with the latest teaching techniques.
3	Facilities available for ELT teaching	No proper facilities are available for ELT teaching;
		Insufficient provision of the latest resources;
		Teachers should arrange their own gadgets for their classes for specific ELT purposes.
4	Professional development and training of ELT teaching	Minimal professional development and training opportunities for ELT teachers are provided;
		Recently, PEELI training was scheduled, but it remained less purposeful due to overcrowded training rooms and resources used during training are not available in schools for those activities during ELT class;
		Mostly, ELT teachers do not have TESOL or TEFL diplomas
5	ELT evaluation	ELT evaluation is unsatisfactory as the examination system demands cramming instead of language proficiency;
		Students spend more time on exam preparation than practicing language skills;
		Lack of reflective mode of practice;
		A more formal examination and evaluation system is required for ELT students.
6	ELT teachers' satisfaction level	Teachers are generally found unsatisfied with the current ELT teaching system in schools;
		Special allowance for ELT teaching, as the other science teachers receive, should be integrated;
		For standard ELT teaching, standard resources should be provided;
		Higher management is cooperative but more needs to plan better and effective ways for ELT teaching;
		Parents' involvement in their children's ELT learning is very disappointing, and they must also educate the parents about the significance of ELT learning.

### 4.5 Questionnaires

The last two tools for data collection, interviews and observations of the English language teachers, showed that teachers in both rural and urban schools had faced many challenges. The most prominent issues and challenges were ELT training, professional qualifications, and lack of infrastructure. Teachers are generally found unsatisfied with the current ELT teaching system in schools. They asked for a special allowance for ELT teaching, similar to integrating other science teachers, and for providing standard resources for ELT teaching. They also pointed out that higher management is cooperative but needs to plan better and more effective ways for ELT teaching. Moreover, parents' lack of involvement in their children's ELT learning is very disappointing, and it is necessary to educate the parents about the significance of ELT learning”.

For the data collection to achieve better results about ELT challenges for the teacher, the questionnaires were prepared accordingly and distributed among two hundred students enrolled in selected schools at the elementary level. All students responded well to the questionnaires, and the ratio of their responses remained one hundred percent. However, some questionnaires were not completed well. For this purpose, their class teachers helped them understand the English content after making its Urdu translation, and then students responded to those questions. The results from the collected data identified the significant themes after analyzing the data are shown in Table 6 in the following.

**Table 6:** Analysis of themes collected from questionnaire data.

Sr.#	Analysis Themes	Description
1	Activities in the classroom	Minimal activities; Teachers in urban and private schools use lessons based on a few AV aids to make them attractive.
2	Teaching Methods/Approaches	Usually, traditional and boring teaching methods are adopted by teachers without any innovation or creativity; Cramming for examinations is focused not on language learning as a skill.
3	Availability and use of ELT Facilities	Minimal facilities in ELT classrooms have been provided; ELT teaching is fully endorsed with technologies.
4	Use of local language in class: Urdu or Punjabi	The students feel comfortable and easy-going with their local language instead of English; Throughout, instructions in English are not fully understood by them.
5	Impression and perception of teachers	The students like their teachers and respect them very well; However, teachers with more creative and innovative teaching methods are their favorites.
6	Overall Feedback	Most of the students were shy and reluctant to give feedback about their teachers' style of teaching and personality; They were not fully aware of the importance of ELT learning for their future.

### 4.6 Learners’ response to questionnaires

The students’ eager participation during the questionnaire session and their collected responses indicate their desire to learn English. To some extent, they also demonstrate awareness of current developments in ELT teaching and learning.

According to Table 7, the majority of students (97 out of 200) agreed that using English conversation in class helps them study English more effectively, as shown in the table above. However, among the 200 respondents, 86 did not agree with this statement and opposed it. Only seventeen students remained neutral and did not express a clear opinion.

**Table 7:** Using English conversations in class helps me study English more effectively.

	Rubrics	Frequency	Cumulative Percent	Percent	Valid Percent
Neutral	SD	33	16.5	16.5	16.5
	D	53	43.0	26.5	26.5
	N	17	51.5	8.5	8.5
	A	48	75.5	24.0	24.0
	SA	49	100.0	24.5	24.5
Total		200		100.0	100.0

According to Table 8, 87 students agreed with the statement that *teachers have command over the subject & use of correct pronunciation*. While among the 200 respondents, 85 did not agree with this statement and opposed it. Only twenty-eight students remained neutral and undecided.

**Table 8:** Teachers have command over the subject & use of correct pronunciation.

	Rubrics	Frequency	Cumulative Percent	Percent	Valid Percent
Neutral	SD	33	16.5	16.5	16.5
	D	52	42.5	26.0	26.0
	N	28	56.5	14.0	14.0
	A	61	87.0	30.5	30.5
	SA	26	100.0	13.0	13.0
Total		200		100.0	100.0

The results obtained from Table 9 indicated that 93 students agreed with the statement that *teachers employ group/pair work techniques*. However, among the 200 respondents, 96 students disagreed and opposed this statement. Only eleven students remained neutral, indicating their indecisiveness.

**Table 9:** Group/pair work techniques used by teachers.

	Rubrics	Frequency	Cumulative Percent	Percent	Valid Percent
Neutral	SD	43	21.5	21.5	21.5
	D	53	48.0	26.5	26.5
	N	11	53.5	5.5	5.5
	A	53	80.0	26.5	26.5
	SA	40	100.0	20.0	20.0
Total		200		100.0	100.0

The results from Table 10 indicate that a majority of the students, specifically 133, agreed that *the use of technology is emphasized in improving listening and speaking skills in the classroom*. Conversely, among the 200 respondents, only 49 disagreed with and opposed this statement. A total of eighteen students remained neutral, indicating their lack of a definitive opinion.

**Table 10:** The use of technology emphasized improving listening and speaking skills in class.

	Rubrics	Frequency	Cumulative Percent	Percent	Valid Percent
Neutral	SD	21	10.5	10.5	10.5
	D	28	24.5	14.0	14.0
	N	18	33.5	9.0	9.0
	A	86	76.5	43.0	43.0
	SA	47	100.0	23.5	23.5
Total		200		100.0	100.0

Based on the results presented in Table 11, it is evident that a majority of the students, specifically 132, agreed with the statement that *the classroom environment is conducive to learning English*. Conversely, out of the 200 respondents, only 45 expressed disagreement and opposed this statement. A smaller group of twenty-three students remained neutral, indicating their lack of a definitive opinion.

**Table 11:** The classroom environment is conducive to learning English.

	Rubrics	Frequency	Cumulative Percent	Percent	Valid Percent
Neutral	SD	12	6.0	6.0	6.0
	D	33	22.5	16.5	16.5
	N	23	34.0	11.5	11.5
	A	80	74.0	40.0	40.0
	SA	52	100.0	26.0	26.0
Total		200		100.0	100.0

### 4.7 Teachers’ questionnaire results and analysis

For the teachers’ questionnaire, a total of eight teachers were randomly selected from both rural and urban schools containing both public and private institutions. All the selected teachers were ELT instructors in their respective schools, and an equal number of male and female teachers were included to ensure gender balance in this study. The participation rate among the teachers was 100 %, as all eight teachers returned the questionnaires within the designated timeframe. The subsequent figures present the results compiled using SPSS software and MS Excel programs.

**Fig. 3:**
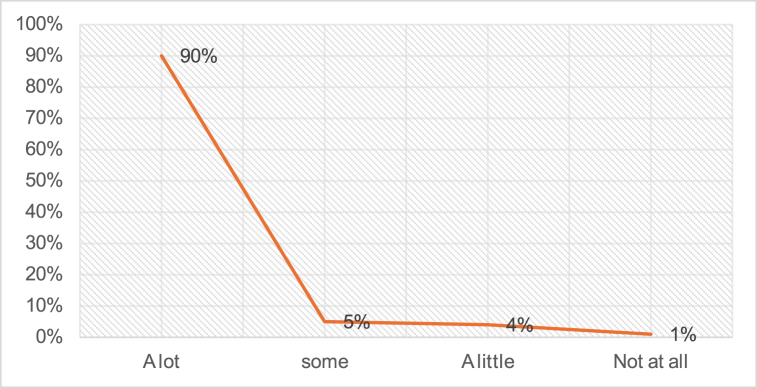
Description of the classrooms.

According to Fig. 3, approximately 90 % of the teachers expressed their agreement with the statement *that classrooms are overcrowded, which is accurate*. In comparison, a small percentage of teachers opted for alternative options; only 5 % were somewhat in favor, and 4 % expressed a mild concern. Surprisingly, only 1 % of the 26 teachers indicated that overcrowded classrooms were not a challenge for them.

**Fig. 4:**
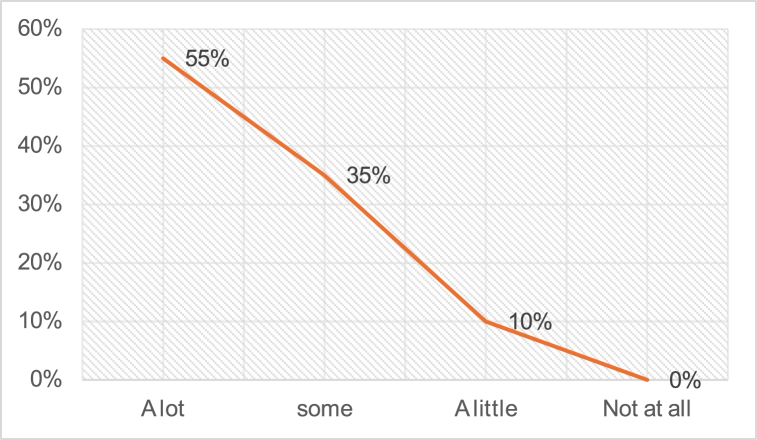
Review the previous knowledge of students.

Fig. 4 shows that around 55 % of the teachers expressed a preference for *asking questions to review students’ previous knowledge.* In contrast, a minority of teachers chose the other options; only 35 % favored asking some questions, and 10 % preferred asking a few questions. Interestingly, none of the 26 teachers indicated that they did not ask questions at the beginning of their class.

**Fig. 5:**
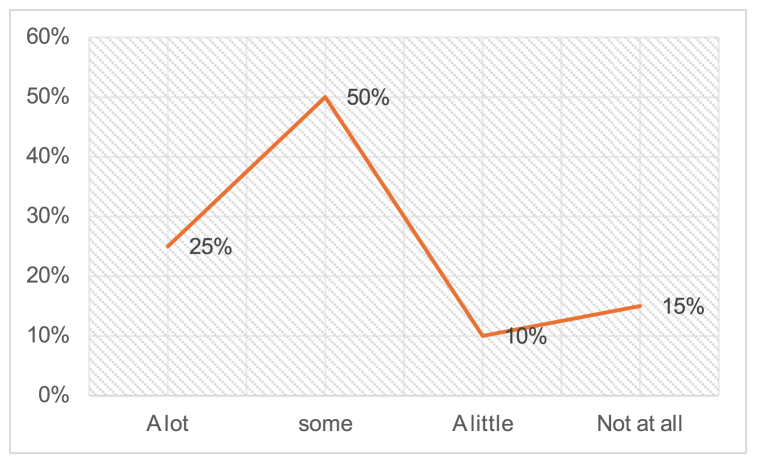
Speak English with students in the class.

In terms of teacher preferences, Fig. 5 indicates that speaking English with students in the class was favored by about 50 % of the teachers. In contrast, a considerable number of teachers, around 10 %, expressed a preference for speaking English in class, while approximately 15 % showed a slight inclination towards it. Interestingly, 25 % of the eight teachers admitted to not speaking English in their classes.

**Fig. 6:**
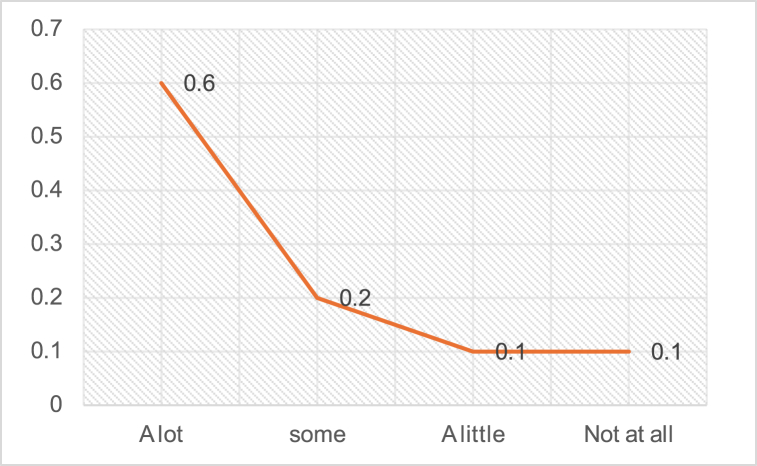
Description of professional training for Teaching English.

Fig. 6 highlights that about 60 % of the teachers favored the idea of having *any professional training for Teaching English that they have professional training for ELT teaching*. In contrast, approximately 20 % of teachers expressed a preference for professional training, while 10 % showed a slight inclination towards it. Notably, 15 % of the 26 teachers stated that they do not have professional training in ELT teaching.

**Fig. 7:**
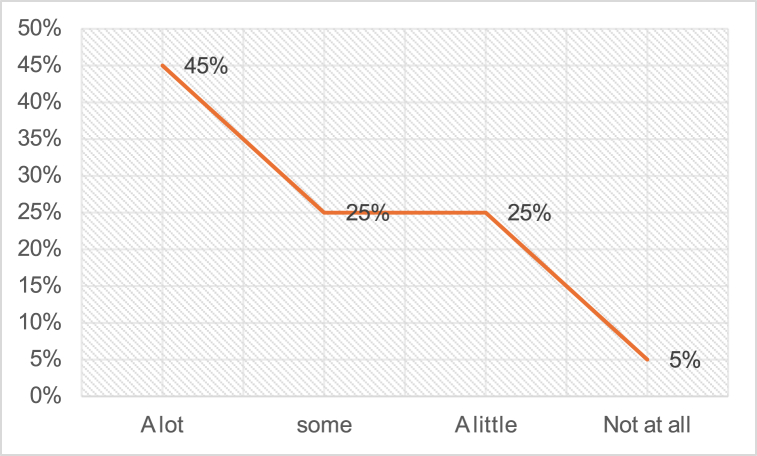
Use of AV aids during teaching Explanation.

Based on Fig. 7, it can be observed that approximately 45 % of the teachers indicated their preference for incorporating visual and audio aids into ELT instruction. In comparison, a smaller percentage of teachers, 25 %, expressed a preference for using some AV aids in the classroom, while another 25 % showed a slight inclination towards AV aid usage. Interestingly, only 5 % of the participants stated that they do not utilize any AV aids for ELT teaching.

Detailed data results and their analysis have been presented through four stages for data collection and description in this study, such as observation, interviews, and questionnaires from students and teachers to identify the challenges in ELT teaching and learning in selected schools. The results and analysis from the collected data spotlighted numerous issues and challenges, such as lack of professional qualification, unavailability of latest technology tools, teachers’ training, lack of furnished classrooms to meet the language study requirements, etc., that the ELT teachers, students, and their institutions have faced. The primary issues and challenges identified during this study are the ones that determine the quality of ELT teaching, learning, and practicing within and outside classrooms.

Based on the collected data, the most frequent challenges faced by ELT teachers and students in Pakistan, particularly in Punjab's rural and urban schools, include inadequate teacher training and professional qualification, overcrowded classes, limited access to up-to-date technology tools, and unfamiliarity with innovative teaching methods. It is worth noting that some exceptional cases were observed in private schools.

According to Fig. 6, ELT teachers in central districts of Punjab need professional training in the latest technologies used in developed countries, as well as subject development courses such as TESOL (Teachers of English to Speakers of Other Languages), TEFL (Teaching of English as Foreign Language), and PEELI (Punjab Education and English Language Initiative). This training will enhance their ability to integrate technology with teaching in their classrooms [39,40]. To address this need, specific professional courses or certifications related to ELT teaching should be added as prerequisites to the qualifications of teachers involved in ELT teaching courses at all levels throughout the country. This will help alleviate the challenges of professional incompetency and address the lack of expertise in ELT-specific issues within the ELT teaching environment.

### 4.8 ELT teachers’ professional development opportunities

The study findings recommend that ELT teachers should acquire additional professional qualifications to enhance their skills. The School Education Department, in collaboration with the Government of Punjab, is actively addressing challenges and issues ELT teachers face in their professional development. Furthermore, teachers without professional qualifications should be provided with specific in-service refresher courses and workshops and encouraged to pursue professional qualifications. Many weekends, afternoons, distance learning, and online resources and institutions are available throughout the country, providing in-service teachers with opportunities to acquire further professional qualifications. In Punjab, three prominent universities offer in-service qualifications for teachers through distance learning.

Allama Iqbal Open University is a prominent institution that offers various distance, regular, and blended learning programs to meet ELT-related requirements. The University of Education is another notable institution providing evening teacher training classes through various government elementary colleges (GECs). Thirdly, COMSATS University, which also launched its Virtual Campuses recently as Virtual University had been providing. Finally, the Directorate of Staff Development (DSD) in Lahore offers in-service teachers extensive PEELI workshops and training [41,42]. In addition to these government institutions, numerous private, accredited, and highly professional institutions offer professional development courses and certifications for ELT teachers. For instance, private entities such as Bacon House University, Care Foundation, and several other institutions and organizations offer ELT professional development programs tailored to accommodate teachers’ inflexible schedules.

## 5 Conclusion

This study investigated instructional practices and primary challenges that English Language Teaching teachers have faced at the elementary level in Punjab, significantly influencing ELT teaching quality. It further provides evidence from the classroom practices to highlight to what extent the educators' instructional practices in selected schools offer quality English language teaching. After comprehensively analyzing the findings and substantial discussion, the researchers have divided the challenges into three categories. First, at the instructional level, teachers should be equipped with the latest tools, techniques, and methods for teaching ELT at the public elementary school level. Second, at the professional development level, teachers' professional qualifications should not be compromised during the hiring process, and in-service training could help overcome the ELT classroom challenges in Pakistan. Third, addressing learners' needs is where the instructional practices and pedagogical techniques should be harmonized with the learning needs of the students. All three categories of challenges need to be seriously addressed for sustainable and effective change. Many students expressed concerns during interviews and observations about teachers’ instructional practices within ELT classrooms. Furthermore, the present study highlights the need to improve ELT teaching quality and overcome the challenges faced by ELT teachers challenges.

## 6 Recommendations

Based on a detailed analysis of the qualitative and quantitative data and discussion conducted, the researchers suggest the following recommendations for future research and English language teaching improvement at the elementary level in developing areas of rural Punjab, Pakistan.

i. Establishment of Standardized Criteria for ELT Teachers' Induction:

Although subject-based teachers have been hired at the secondary level in Punjab since 2009, the situation at the primary and elementary levels is concerning. Many examples were found during this study where teachers of non-English language backgrounds and degrees were teaching language classes. Urgent action is needed to address this practice and ensure the appointment of English language teachers with relevant degrees. A strict criterion must govern the induction of new ELT educators hiring. These criteria should be based on professional qualifications specifically relevant to ELT, ensuring a comprehensive and standardized approach to the recruitment process.

ii. 2. *Continuous Professional Development for In-Service ELT Teachers:*

Subject experts should organize and implement specialized professional development training to handle the issue more rapidly. These initiatives should target in-service ELT educators with limitations in obtaining further professional qualifications. These initiatives are designed to enhance and modernize their teaching methodologies and adept utilization of resources throughout ELT sessions.

iii. 3. *Addressing Classroom Overcrowding:*

To make the ELT teaching process smooth and more interactive, serious efforts must be directed toward mitigating the issue of classroom overcrowding. It is recommended that the standard class size should be between 25 and 30 students only to allow educators to interact individually with each student, monitor their language learning progress, and ensure efficient teacher-student interaction.

iv. 4. *Integration of Technological Resources in ELT:*

It is necessary to align existing teaching methods and approaches to informational and communication technologies (ICT) recourses to cope with the emerging technologies era and their integration into education. Considering the crucial role of ICT in contemporary language education, serious efforts should be made to ensure the proper use and equitable distribution of technological resources among ELT staff and institutions. This strategic approach aims to harness the full potential of specialized tools in enhancing the utility of ICTs in language education.

v. 5. *Fostering a Culture of Knowledge and Experience Sharing:*

The interview data analysis revealed a lack of staff exchange and knowledge-sharing practices among ELT instructors. The education department and school heads should plan to exchange English language faculty to promote a knowledge-sharing culture so that in-service ELT educators can get diverse experiences from their peers. Establishing forums, workshops, or platforms conducive to exchanging insights and pedagogical expertise among ELT practitioners is essential.

## Data availability

No secondary data is associated with this study. For primary data sources, information will be provided on request.

## Ethical consent

A written ethical consent has been taken from the ethical committee at the University of Sahiwal, Pakistan, because the corresponding author is a lecturer at the university.

## CRediT authorship contribution statement

**Muhammad Imran:** Writing – review & editing, Writing – original draft, Visualization, Validation, Software, Resources, Investigation, Formal analysis, Data curation, Conceptualization. **Norah Almusharraf:** Writing – review & editing, Project administration, Methodology, Funding acquisition, Formal analysis. **Mohamed Sayed Abdellatif:** Writing – review & editing, Visualization, Validation, Software, Resources, Formal analysis. **Abdul Ghaffar:** Writing – original draft, Validation, Software, Formal analysis, Data curation.

## Declaration of competing interest

The authors declare that they have no known competing financial interests or personal relationships that could have appeared to influence the work reported in this paper.
