# Supplementary material for: Teachers’ perspectives on effective English language teaching practices at the elementary level: A phenomenological study
Source: Heliyon. 2024 Apr 7;10(8):e29175. doi: 10.1016/j.heliyon.2024.e29175 (PMC11016971; doi:10.1016/j.heliyon.2024.e29175)
Supplement: Multimedia component 1 [file mmc1.docx]

**Observation Sheet/Checklist for ELT Class Visits**

Observer: [Name] Date: [dd-mm-yyyy]

**1. Classroom Management:**

- [ ] Clear rules and expectations are established.

- [ ] Smooth transition between activities is evident.

- [ ] Student engagement and participation are effectively managed.

- [ ] The physical layout supports effective classroom management.

**2. Teaching Methods/Approaches:**

- [ ] Diverse teaching methods are employed.

- [ ] Instructional approaches cater to different learning styles.

- [ ] Use of technology and multimedia is observed.

- [ ] Opportunities for student collaboration and interaction are facilitated.

**3. Availability of ELT Facilities:**

- [ ] Adequate teaching materials are accessible.

- [ ] Audio-visual aids are functional and utilized effectively.

- [ ] Classroom setup supports language learning activities.

- [ ] Availability of resources for varied language skills development.

**4. Activities and Interactions in ELT Teaching:**

- [ ] Varied and engaging activities are incorporated.

- [ ] Teacher-student interactions are respectful and supportive.

- [ ] Student-student interactions foster language use.

- [ ] Language skills are integrated into activities effectively.

**5. Other Observed Significant Challenges Faced by ELT Teachers:**

- [ ] Notable challenges with student engagement.

- [ ] Issues related to classroom dynamics.

- [ ] Challenges in adapting to diverse learning needs.

- [ ] Any other challenges observed (please specify).

Additional Comments:

---------------------------------------------------------------------------------------------------------------------------------------------------------------------------------------------------------------------------------------------------------------------------------------------------------------------------------------------------------------

Note: This observation sheet is designed to capture key aspects during ELT class visits, including classroom management, teaching methods, facility availability, activities, interactions, and challenges faced by ELT teachers.

**Questionnaire for Teachers**

Dear Educators,

Your feedback is invaluable for refining the English language education experience. Please share your perspectives on the following aspects. Please rate each statement on a scale of 1 to 4, where 1 represents "Not at all," 2 represents "A little," 3 represents "Some," and 4 represents "A lot."

**1. Do you a*sk questions to review students’ previous knowledge in language class?***

1. Not at all

2. A little

3. Some

4. A lot

***2. Do you speak English with students in class?***

1. Not at all

2. A little

3. Some

4. A lot

***3. Do you have any professional training for Teaching English?***

1. Not at all

2. A little

3. Some

4. A lot

***4. Use of AV Aids during language teaching***

1. Not at all

2. A little

3. Some

4. A lot

***5. Are you satisfied with the facilities available for language teaching?***

1. Not at all

2. A little

3. Some

4. A lot

Additional Comments:

Feel free to share any additional insights, comments, or suggestions related to your experiences in teaching English.

Thank you for your valuable input. Your perspectives contribute to the ongoing improvement of English language education.

**Questionnaire for students**

Dear Participants,

Thank you for taking the time to provide your valuable insights. Your feedback is crucial in enhancing the quality of English language learning experiences. Please respond to the following statements by indicating your level of agreement on a scale of 1 to 5, where 1 represents "Strongly Disagree" and 5 represents "Strongly Agree."

**1. *The use of English conversation in class helps me study English more effectively.***

1. Strongly Disagree

2. Disagree

3. Neutral

4. Agree

5. Strongly Agree

**2. *Teachers have command over the subject & use of correct pronunciation.***

1. Strongly Disagree

2. Disagree

3. Neutral

4. Agree

5. Strongly Agree

***3. Group/pair work techniques are used by teachers***

1. Strongly Disagree

2. Disagree

3. Neutral

4. Agree

5. Strongly Agree

**4. *Use of technology is emphasized in improving listening and speaking skills in the class.***

1. Strongly Disagree

2. Disagree

3. Neutral

4. Agree

5. Strongly Agree

***5. Classroom environment is conducive to learning English***

1. Strongly Disagree

2. Disagree

3. Neutral

4. Agree

5. Strongly Agree

Additional Comments:

Please feel free to provide any additional comments or suggestions regarding your experience in learning English in the classroom.

**Interview Questions for Teachers**

1. What are the most significant challenges you face in ELT teaching?

2. Can you outline the key qualities you believe define an effective ELT teacher?

3. Briefly describe the essential facilities for effective ELT teaching.

4. How do you prioritize and engage in ongoing professional development and training in ELT teaching?

5. How do you approach the evaluation of English language teaching effectiveness in your classroom?

6. On a scale of 1 to 10, how satisfied are you with your current role as an ELT teacher, and what factors contribute to your satisfaction level?
